# Implementation of external quality assessment of microscopy for improved parasite detection and confirmatory diagnosis of malaria in Tanzanian Military health facilities

**DOI:** 10.1186/s13104-020-05290-0

**Published:** 2020-09-18

**Authors:** Akili K. Kalinga, Saidi Mgata, Reginald A. Kavishe, Lucas Mahikwano, Lucky Temu, Christopher Mswanya, Charles Mwanziva, George Amoo, Edwin Kamau, Brian Vesely, Deus S. Ishengoma

**Affiliations:** 1 National Institute for Medical Research, Dar es Salaam, Tanzania; 2 Kilimanjaro Christian Medical University College, Moshi, Tanzania; 3 Henry Jackson Foundation Medical Research International, Dar es Salaam, Tanzania; 4 Tanzania Peoples Defense Forces, Dar es Salaam, Tanzania; 5 Forgyn Health Systems Consultants, Washington, DC USA; 6 Walter Reed Army Institute of Research, Washington, DC USA; 7 Faculty of Pharmaceutical Sciences, Monash University, Melbourne, Australia; 8 Harvard T.H Chan School of Public Health, Harvard University, Boston, MA USA

**Keywords:** Malaria, Microscopy, External quality assessment, Proficiency testing, Total quality index, Military health facility, Tanzania

## Abstract

**Objective:**

Good quality microscopy is critical for accurate detection and confirmation of malaria parasite infections. Microscopy relies on the skills of technicians to prepare and read slides, high quality reagents, and a good program of internal and external quality control (EQA), which are lacking in most malaria endemic settings. This study was undertaken between January 2016 and December 2018 to pilot an EQA of microscopy for improved diagnosis of malaria and patient care in Tanzanian Military health facilities.

**Results:**

Of all blood smears crosschecked (n = 4000) at baseline, only 38.5% were incorrectly diagnosed by laboratory staff with false positive and negative rates of 46.7% and 16.4%, respectively. During the implementation of EQA, false positive and negative results decreased due to increased quality index of slide preparation and reading through supportive supervision, and retraining of laboratory personnel. There was a gradual increase of quarterly and annual total quality index for all laboratories, from 60% in 2016 to 78% in 2017 and 90% in 2018. The mean proficiency testing performance scores also increased from 75% in 2016 to 82% in 2017 and to 90% in 2018. Poor blood smear preparation and staining contributed to high false positive and negative rates while EQA helped in improvement of diagnostics.

## Introduction

Prompt parasitological detection and confirmation of parasite infections is recommended as an important pillar of malaria case management for improved care and treatment of febrile patients [1, 2]. Despite recent introduction of rapid diagnostic tests, good quality microscopy is still considered a reference test for malaria diagnosis [3, 4]. However, the effectiveness of malaria microscopy depends on maintaining a high level of competence and performance of laboratory staff, ensuring good-quality reagents, proper preparation and staining of blood smears and regular internal and external quality assessment, which are currently lacking in most malaria endemic countries [4, 5].

The World Health Organization (WHO) recommends for malaria endemic countries to implement a comprehensive external quality assessment (EQA) to ensure the quality of malaria diagnosis by microscopy [4, 6]. The EQA describes a method that allows testing conducted by a laboratory, testing site or individual user to be compared to that of a source outside the laboratory [7]. Traditionally, EQA programs focus exclusively on reading accuracy. However, the accuracy of microscopy is also critically dependent on the quality of blood smear preparation and staining procedures because poorly prepared and/or stained blood films directly reduces reading accuracy regardless of reading skill. Therefore, EQA through randomized slide re-checking of samples, and proficiency testing of laboratory staff are important malaria microscopy quality improvement program’s (QIPs) that contribute to improvement of diagnosis, and ultimately the quality of patient care [8, 9].

Cross-checking is an important component of effective EQA that indicates whether a laboratory is providing accurate results, and can detect major deficiencies in laboratory performance due to low competence of staff, poor equipment, poor reagents, poor infrastructure or poor work practices [4]. Cross-checking malaria slides for quality of smear prepared by facility microscopists and readers’ results is a standard EQA process recommended by the WHO for quality improvement [7]. Proficiency testing is another important aspect of EQA, which is implemented when a reference laboratory sends stained blood smear samples with known parasitaemias to testing laboratories for assessing and reading to determine the accuracy of laboratory staff. Upon reading and submission of the results, the reference laboratory gives feedback about the correct and incorrect reading results by individual laboratory staff, and overall laboratory performance. When under performance of routine malaria microscopy slide preparation and reading accuracy at health facilities is observed, that calls for interventions to improve the quality and performance of the laboratory procedures and training [9, 10]. Such interventions might include supportive supervision coupled with onsite training by supervisors from higher level laboratory [6, 11].

In Tanzanian military health facilities (MHFs), microscopy is commonly used due to shortage of rapid diagnostic tests (RDTs) as a result of unsustainable costs to meet the high demand of large influx of military recruits at the camps. Unfortunately, due to lack of expertise and financial resources, there is no microscopy EQA scheme. In this study, through Walter Reed Army Institute of Research of US, we piloted an EQA program in MHFs of Tanzania Peoples Defense Forces (TPDF) to provide objective data on the quality of malaria diagnosis.

## Main text

### Materials and methods

#### Study area and populations

The study was done in eight MHFs located in six malaria endemic regions across Tanzania. The MHFs included Bulombora, Chita, Kaboya, Maramba, Mgambo, Msange, Ruvu and Rwamkoma.

The study population included patients admitted (inpatients) and not admitted (outpatients) at MHFs.

#### Study design and period

A cross-sectional study was conducted in selected facilities from January 2016 to December 2018.

#### Sample size and sampling

A total of 16 professional laboratory staff were purposively recruited in QIPs at their respective MHFs. A total of 16,000 slides with thick blood smears from all the laboratories at the MHFs formed a sampling frame. Of these, 20% (n = 3200) were positive of which 65% (n = 2080) were found and collected for assessment. A total of 1920 negative slides were also collected by selecting 10 slides each month using a random sampling method from each MHF in 10 rounds done on quarterly visits. For proficient testing, 2240 slides with standardized expert validated blood smears were available.

#### Training of microscopists

Prior to commencement of the microscopy QIPs at their MHFs and after competency assessment test, 16 microscopists trained as laboratory technicians and assistants who attended standard 2 weeks’ microscopy course at Malaria Diagnostic Centre as per WHO curriculum participated in the study. The microscopists were assessed by three expert microscopists who were taking part in annual proficiency testing.

#### Supply of laboratory equipment, reagents and consumables

Unlike before implementation of QIP, during quarterly site visits when EQA started, as part of the quality improvement program, MHFs were supplied sustainably with high quality malaria diagnostic equipments (microscopes, slide warmers, etc.), reagents (stains and fixatives) and laboratory consumables (slide preparation template, distilled water etc.).

#### Quality control

The smears were read for the first time by microscopists at MHFs. Two experienced expert microscopists performed the second reading as another microscopist performed the third read to break-tie the discordant results of the two assessors.

#### Data collection

*Slide crosschecking* Each quarter, assessors collected from storage boxes, slides that were 2–3 months old. Assessors selected in situ from the laboratory register, all reported positive and 10 negative blood slides each month. Negative slides were selected for re-reading at the reference laboratory, using a representative sampling protocol (Additional file 1) to ensure random distribution from the start, middle and end of working day and from variable days distributed across the month. Thick blood smears were assessed for quality of preparation, staining and reading accuracy. Slides were assessed macroscopically and microscopically on 12 key parameters namely, labelling, not cracked, not fixed, no fungus, no bacteria, no wash, size of smear, thickness of smear, uniformity of smear, correct staining and reading the results (Additional file 2). The assessment aimed at scoring total quality index (TQI) of laboratory as quality performance. The TQI is a combined score (%) from all microscopists for the macroscopic and microscopic 12 parameters of blood smears at MHFs. It is the average score of all microscopists for a month, quarter and year under review.

*Proficiency testing* It was done using malaria slides provided by National Malaria Slide Bank (NMSB). The slides had been read and validated by four blinded National and International experts with WHO certification. During supervision visits, each laboratory staff was assigned to read 20 slides with known parasitaemia of which nine were negatives and 11 were positives with low (< 100 asexual parasites/µl) or high (≥ 100 asexual parasites/µl) parasitaemia. The results for each laboratory staff was compared against known results of the samples then aggregated quarterly for each MHF. The quarterly average performance of proficiency testing was calculated for each participating MHF.

#### Data analysis

The data was managed using Microsoft Excel software and analyzed using STATA software (STATA Inc, TX, USA). Statistical significance was measured at P-value < 0.05. The results of the EQA was reported for 12 parameters using quality index indicators of well performed (over 75%) for more than 9 parameters, fairly performed (50 to 75%) between 6 and 9 parameters, and poorly performed (< 50%) for less than 6 parameters.

### Results

#### Characteristics of items for external quality assessment

Of the 16 laboratory staff assessed, 13 were assistant health laboratory technologists and two were health laboratory technologists. A total of 4000 slides (1920 negative and 2080 positive) were selected for cross-checking while 2240 validated slides were given to laboratory staff for proficiency testing. Proficiency testing was done three quarters later compared to crosschecking that started earlier in quarter one of 2016.

#### Slide crosschecking

**Fig. 1:**
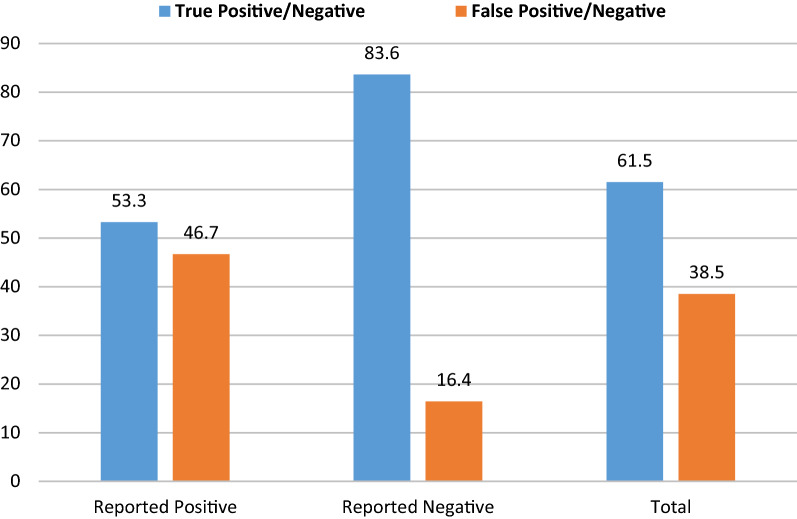
True and false rate of test results among blood smears reported by MHFs at baseline

At the start of the EQA, the baseline data showed that preparation and staining of blood smears were well performed (> 75%) for more than half of parameters assessed. About one quarter of all parameters were performed fairly (between 50 and 75%) while two parameters were performed poorly (< 50%). The smears read by facility staff, 61.5% were correctly diagnosed and 38.5% were incorrectly diagnosed (Fig. 1). For the smears reported as positive, 53.3% were true positive while 46.7% were false positive. With respect to smears reported as negative, 83.6% were true negative while only 16.4% were false negative. The rate of false positive over time decreased with an increase in the TQI of slide preparation; likewise, false negatives increased with a decrease in quality of preparation (Additional files 3 and 4).

**Fig. 2:**
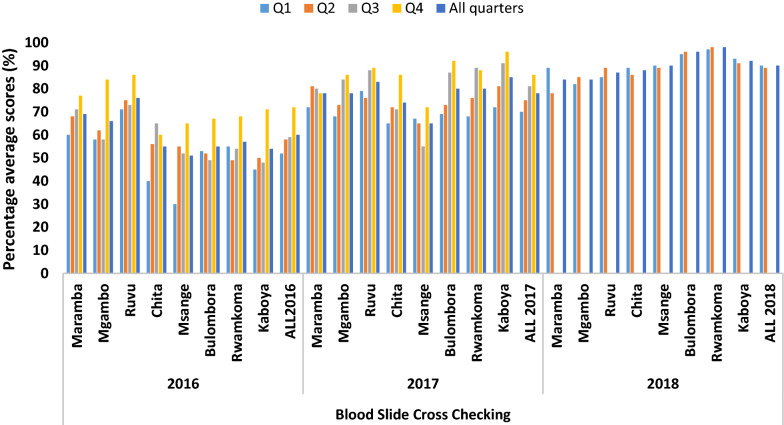
Trends of TQI on cross-checking blood smear by health facilities

Slide cross-checking revealed gradual increase of TQI from 52% in quarter one to 58%, 59% and 72% in quarters two, three and four respectively. Annual mean combined TQI for all studied laboratories increased from 60% in the first year (2016) to 78% in the second year (2017) and 90% in the third year (2018) (Fig. 2).

#### Proficiency testing

**Fig. 3:**
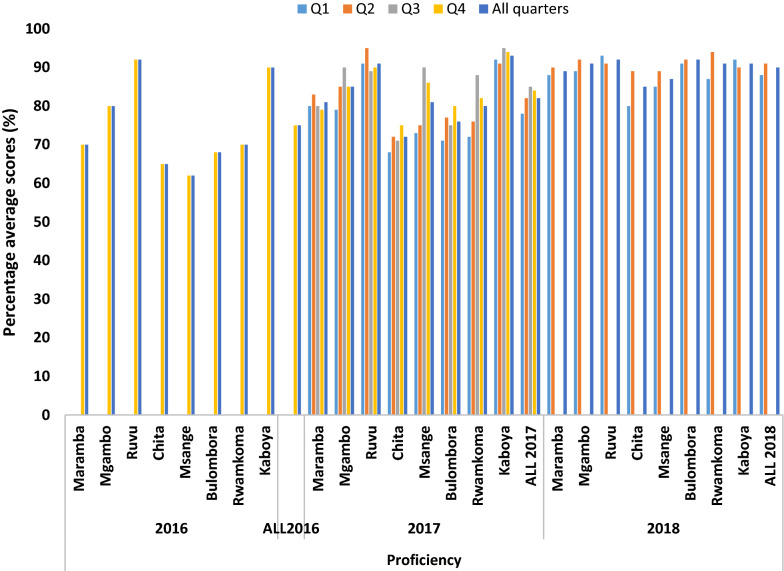
Trends of proficiency testing average scores by health facility over time

After 1 year, the average of proficiency test scores increased from 75% in quarter four in 2006 to 82% in quarter four in 2017. Generally, the mean proficiency testing performance scores showed an increasing trend from 75% in the first (2016) to 82% in the second (2017) and to 90% in the third year (2018) (Fig. 3).

### Discussion

The findings from ten quarterly visits to MHFs demonstrated that supportive supervision contributed to identifying gaps, and through on-site training, a remarkable improvement in the quality of malaria diagnosis by microscopy was achieved. The increased performance was noticed among individual microscopists and the average aggregated results for each of the MHF as described previously [12, 13]. In our view, the reported improvement could also be attributed to provision, and sustaining availability of quality laboratory equipment, reagents and consumables [12]. The observed improvements were apparent in the second quarter after implementing the QIPs during the first quarter, and was maintained in the subsequent quarters and over the three years.

Despite the observed increasing trend of overall laboratory performance for TQI (slide crosschecking) and combined scores (proficiency testing), few MHFs such as Ruvu did not show steady quarterly increasing trends. The reason behind this is the average performance in the final score of the facility being pulled down by low performers among introduced new laboratory staff with less experience in microscopy in the reporting quarters compared to the previous ones when only trained experienced staffs performed well. This was confirmed when newly introduced laboratory staff were re-trained and started to improve their individual scores, which ultimately raised the overall performance of the respective facility [14].

During the first quarter of assessment, the baseline data of combined TQI (from Crosschecking) was found to be 52% lower by 23% compared to 75% of a combined score (for proficiency testing) in the first quarter as well for all MHF. Similarly, the TQI in the first year (2016) was lower by 15% compared to combined proficiency testing scores for all MHFs. The possible explanation for the difference could be due to the effects of on-site retraining of laboratory staff that was conducted after the baseline assessment in the first quarter and before initiation of proficiency testing which started later in the fourth quarter of 2016. That is, by the time laboratory staff were assigned validated slides for proficiency testing, they had acquired reading skills during the initial training and re-training in the previous three quarterly visits.

Generally, a lower (52%) mean preparation TQI for blood smear and high false positive rate (46.7%) at the baseline, suggested that detection of malaria parasites in a poorly prepared and stained smear becomes more difficult and significantly reduces the sensitivity of microscopy. This was verified by re-training laboratory staff and improvements made thereafter. Unlike before implementation of EQA when the false test results were high, we observed improvement in total quality index that was associated with decrease of false positivity rates (graph S3) and vice versa. Likewise, false negatives increased with a decrease in quality of preparation (graph S4). That is, slides which had high quality with respect to preparation with contributed to increased accuracy in detection of the parasite and vice versa. Therefore, the results strongly suggest that poor preparation and staining of blood smear contribute significantly to the high rates of false results. Therefore, EQA programs should address quality of preparations and staining rather than focusing only on microscopists reading skills to ensure high quality of malaria diagnosis and case management. Thus, the overall improvement in the diagnostic services observed in this study was potentially attributed to increased site assessment scores as a result of implemented EQA at MHFs.

## Study limitations

The implementation of supportive supervision was observational in nature making it difficult to isolate the effects of supportive supervision among other parameters in the observed improvement in quality diagnosis.

## Supplementary information

**Additional file 1: Figure S1.** Malaria microscopy external quality control protocol.

**Additional file 2: Figure S2.** Preparation and staining parameters of blood smears on the slide.

**Additional file 3: Figure S3.** A graph showing trend of false positives versus preparation quality.

**Additional file 4: Figure S4.** A graph showing trend of false negatives versus preparation quality.

## Data Availability

The datasets used for this study are available on reasonable request from the corresponding author.

## Abbreviations

EQA: External Quality Assessment
MHFs: Military Health Facilities
TPDF: Tanzania Peoples Defense Forces
TQI: Total Quality Index
QIPs: Quality Improvement Programmes
WHO: World Health Organization
