# Supplementary material for: Implementation of external quality assessment of microscopy for improved parasite detection and confirmatory diagnosis of malaria in Tanzanian Military health facilities
Source: BMC Res Notes. 2020 Sep 18;13:447. doi: 10.1186/s13104-020-05290-0 (PMC7501635; doi:10.1186/s13104-020-05290-0)
Supplement: Supplementary file 1 — Additional file 1: Figure S1. Malaria microscopy external quality control protocol. [file 13104_2020_5290_MOESM1_ESM.pdf]

Additional File 1

MALARIA MICROSCOPY EXTERNAL CROSS-CHECKING QUALITY CONTROL

PROTOCOL

Version 1

October 2014

Contents

PROTOCOL.......... 2

DEFINITIONS.......... 4

DATA ENTRY.......... 8

REPRESENTATIVE SAMPLING PROTOCOL.......... 10

REPRESENTATIVE SAMPLING TABLE.......... 14

DATA ENTRY FORMS.......... 14

- MACROSCOPIC – Positive Blood Films
- MACROSCOPIC – Negative Blood Films
- MICROSCOPIC – Positive Blood Films
- MACROSCOPIC – Negative Blood Films

PROTOCOL

Routine Laboratories

Each month all routine laboratories send:

1. 10 negative blood films selected using the Representative Sampling Table
    a. If there are less than 10 slides examined during the month, then send all negative slides
2. All positive blood films

Reference Laboratory or Designated Cross-checker

1. **Record all results using the ZAMEP Data Entry Form**
2. Score ‘1’ for each correct parameter and ‘0’ for each incorrect parameter

Example

NEGATIVE THICK FILM MACROSCOPIC	Slides										
	1	2	3	4	5	6	7	8	9	10	Total score
Number of slides examined	10										
Labelling – correct - fully and clearly labelled	1	1	0	0	1	1	0	0	0	0	4

10 slides are examined. Slides 1,2,5,6 are correctly labelled and scored ‘1’. The remaining slides are scored zero.

Positive slides

1. Macroscopic
    a. Assess all blood films macroscopically for labelling, same size as template and wash-away.
    b. Assess blood films that are able to be examined for thickness, uniformity, fixation with methanol and cracking due to over-heating.
        i. If a blood film cannot be assessed, score zero.
2. Microscopic
    a. Microscopically examine all slides for staining coloration, stain precipitate, excessive artefacts, bacterial and fungal contamination
    b. Examine all slides for malaria parasites
        i. Read positive slides 100 fields to maximize the detection of mixed-infections
        ii. Identify the species of all positive slides
        iii. If no malaria parasites are detected after 100 fields, then read 200 fields before reporting a blood film as negative (false-positive).

Negative slides

1. Macroscopic
    a. Assess all blood films macroscopically for labelling, same size as template and wash-away.
    b. Assess blood films that are able to be examined for thickness, uniformity, fixation with methanol and cracking due to over-heating.
        i. If a blood film cannot be assessed, score zero.
2. Microscopic
    a. Microscopically examine all slides for staining coloration, stain precipitate, excessive artefacts, bacterial and fungal contamination
    b. Separate negative blood films into 2 groups:
        i. Slides to be examined – slides of sufficient quality to examine accurately
        ii. Slides to be scanned – poor quality slides that cannot be accurately examined
    c. Examined slides
        i. Read each slide for 100 fields
        ii. Record the number of slides confirmed as negative
    d. Scanned slides
        i. Scan each slide briefly for obvious parasitaemia
        ii. If a negative slide is found to be positive, record as a false-negative
        iii. Record all slides as not able to be accurately examined

DEFINITIONS

Macroscopic

1. Labelling

   Blood films must be fully and clearly labelled with the patient ID and the date.

2. Size

   Thick blood films must be circular and approximately the same size as the template. A +/-5% tolerance is acceptable.

3. Wash-away

   Holes, large or small, in the thick blood film.

   Small holes (A) are commonly caused unclean microscope slides (grease, residual immersion oil).

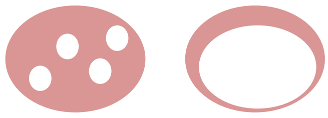

A B

   Large holes (B) are commonly caused by:

   a. Too thick a blood film
   b. Wash buffer directly applied to the thick film

   The Cross-checker must determine if there is sufficient thick blood remaining to assess the film for thickness and uniformity.

   Example – Blood film A can be further assessed, blood film B cannot be further assessed.

4. Thickness

   The density of the thick blood film is consistent with a correct volume of blood sample used to prepare the blood film.

5. Uniform

   The blood is spread evenly (homogenously) throughout the thick film

6. No methanol fixation / Not cracked

   No fixation or cracking – even minor.

Examples:

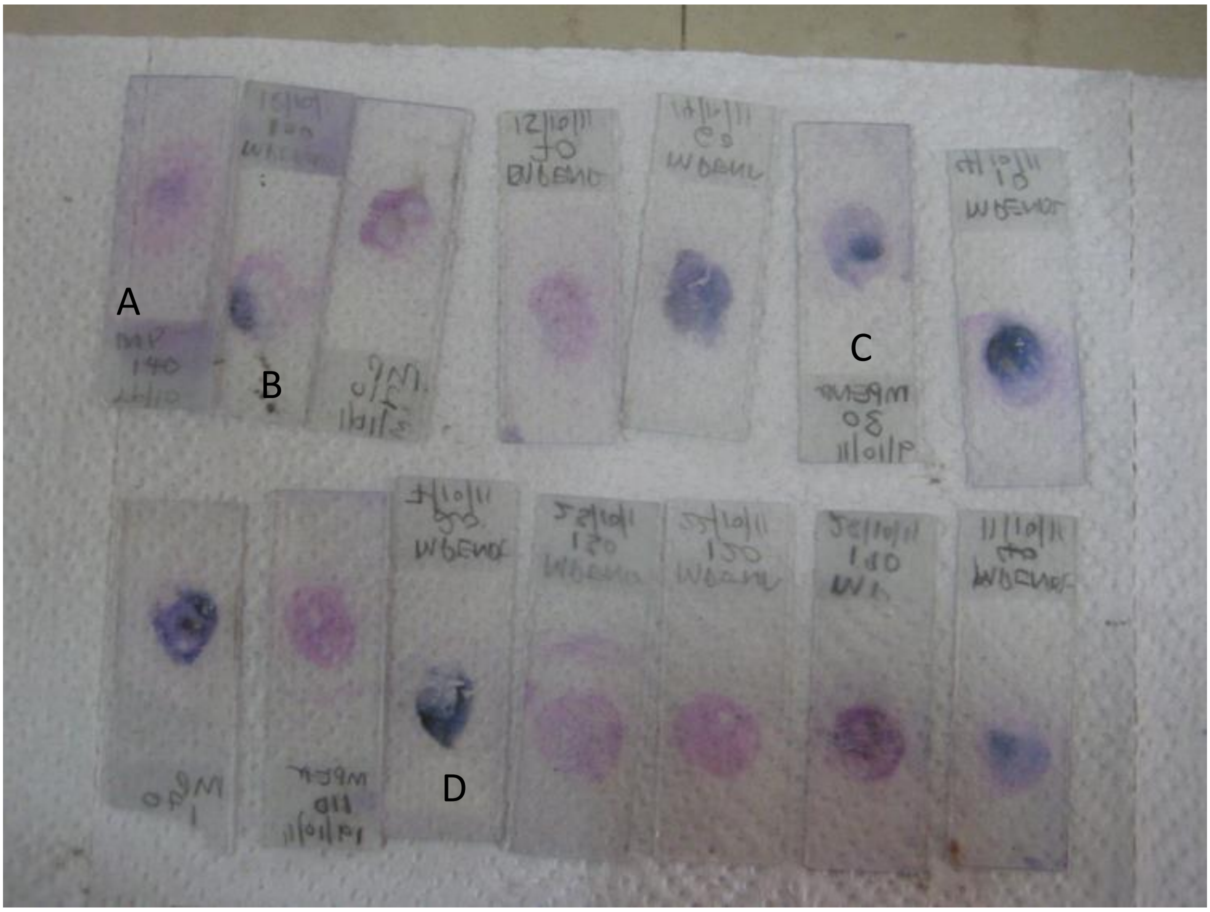

A Too thin

B Wash away

C Not uniform

D Too thick

7. Staining

Staining quality of malaria parasites cannot be assessed for negative blood films. The definition used is ‘the blood film is not blue’.

In the above image there are two rows of 7 thick blood films.

a. 1st row (top) – from left slides 2, 5,6, 7 are blue – incorrect, score 0
b. 2nd row – from left slides 1, 3, 7 are blue – incorrect, score 0.

Microscopic

1. No stain precipitate

   Blood films must contain no stain precipitate

   a. The most common cause of stain precipitate is staining slides with Giemsa that has not been diluted immediately before use. 1:10 diluted Giemsa stain contains precipitate within 15 minutes after dilution, and significant precipitate after 30 minutes.
   b. Blood films containing stain precipitate is are almost always stained blue. Stain precipitate contains large amounts of eosin so that old stain changes from Giemsa to Methylene Blue.

2. No excessive artefacts

   General debris. The stained blood film background should be should not contain large amounts of debris that could be misidentified as 'malaria parasites', or interfere with the detection of malaria parasites.

   Bacterial and fungal contamination are assessed separately.

   a. The critical term is 'no excessive'.
      i. Most stained blood films contain some artefacts (platelet debris etc).
      ii. Blood films contain excessive artefacts if:
         - prepared using unclean slides
         - and/or the stain is prepared using unclean glassware
         - and/or stained with buffer/stain prepared using unclean water

3. No bacterial contamination

   This specifically refers to the presence of bacteria.

4. No fungal contamination

   This specifically refers to the presence of fungus.

   a. The most common cause of fungal contamination is using slides that have become etched with fungus. Fungus contaminated microscopy glass slides should never be used.

5. Weak and strong positive slides

   For reporting:

   a. Positive blood film – defined as a blood film containing malaria parasites detected by the Cross-checker.
   b. Negative blood film – defined as a blood film containing no malaria parasites after 100 fields have been examined by the Cross-checker.

For <u>analysis</u>:

False positive and negative blood films need to be separated into 'weak' and 'strong' for meaningful analysis.

a. Strong positive – defined as ≥ 5 parasites/100 fields.
b. Weak positive – defined as 1-4 parasites/100 fields

Rationale:

Finding 1-4 parasites/100 fields is largely dependent on chance.

Example: The following blood film was examined 5 times;

Reading	*Number of fields*	*Number of parasites*
1	*100*	*1*
2	*100*	*0*
3	*100*	*1*
4	*100*	*0*
5	*100*	*2*

The variation is within normal statistical variation. The following can occur by chance alone:

Routine Laboratory	*Reference Laboratory*	*Report*
Reading 2 (0)	*Reading 4 (0)*	*True negative*
Reading 1 (1)	*Reading 3 (1)*	*True positive*
Reading 1 (1)	*Reading 2 (0)*	*False positive*
Reading 2 (0)	*Reading 3 (1)*	*False negative*

In contrast if there are ≥ 5 parasites/100 fields then every set of 100 fields should contain at least 1 parasite. There should therefore be agreement between the routine and reference laboratories.

DATA ENTRY

Screen 1 – Welcome Page

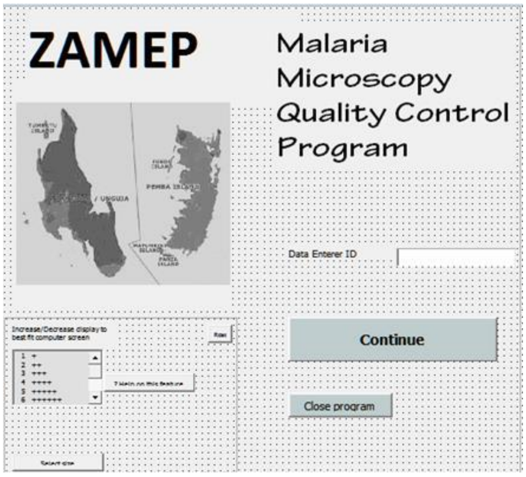

Data Enter ID – this is the name of the person entering the data.

This may not be the same person who performed the QC.

Screen 2 – Laboratory and Sample Details

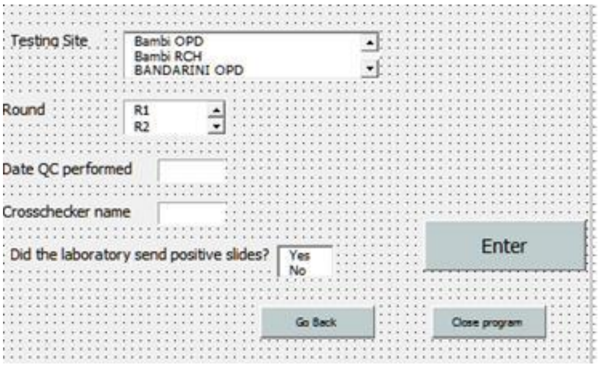

Cross-checker name – this is the name of the person who performed the Cross-checking QC

Screen 3 – Positive Slides Macroscopic & Screen 5 Negative Slides Macroscopic

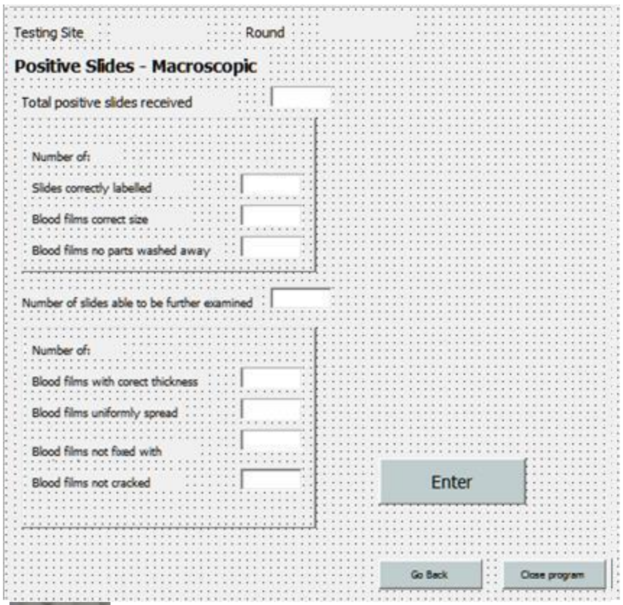

Screen 3 will not be shown if there are no positive slides sent to the reference laboratory.

First box

All slides must be examined for labelling, size and parts washed away.

Number of slides able to be further examined.

Example of a blood film that cannot be further examined macroscopically.

It is not possible to assess thickness, uniformity or cracking.

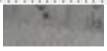

Note – the blood film can be examined microscopically for staining, precipitate, artefacts bacteria and fungus. It cannot be assessed for reading accuracy. See screens 4 and 6.

Important – some blood films will have some parts washed away but have sufficient blood film remaining to be further assessed.

Example – 10 slides are received. 3 have significant wash away and cannot be further macroscopically assessed. 2 have parts washed away but can be further macroscopically assessed. Enter 3.

Second box

This records the number of correct slides.

Important – if a blood film cannot be assessed then score as zero.

Example – 10 slides are received and 3 have significant parts washed away. Therefore only 7 can be further examined. If 4 of these slides have the correct thickness, then enter 4.

Screen 4 – Positive Slides Microscopic & Screen 6 Negative Slides Microscopic

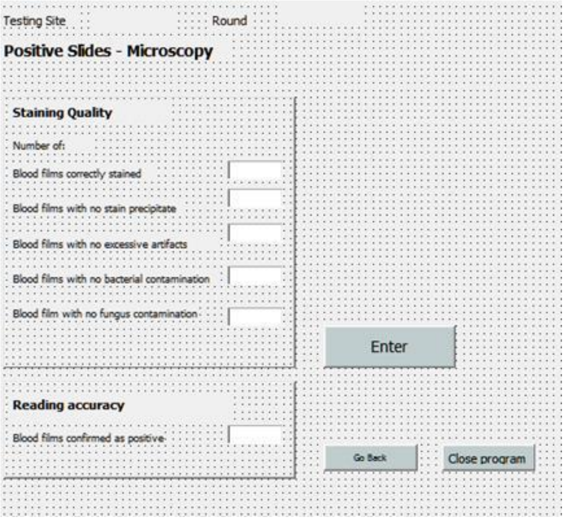

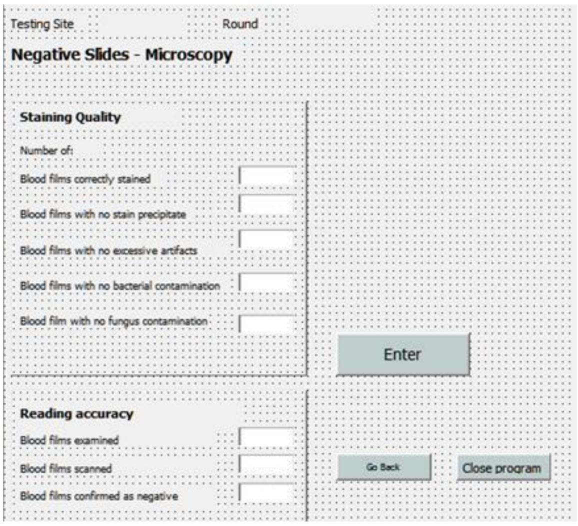

Screen 4 will not be shown if there are no positive slides sent to the reference laboratory.

First box

All blood films should be assessed (even significant wash away slides).

If a slide has no blood film (unusual but possible) then score as zero.

Second box

**Positive slides**	**Negative slides**
Examine all positive slides	Only examine acceptable quality negative slides.<br>-score unreadable slides as zero<br><br>Scan unreadable negative slides but report as either:<br>- false negative<br>- not readable
Because these slides were reported as positive malaria parasites should be detected even if the slides are poor quality.	

Enter the number of negative slides examined and scanned.

REPRESENTATIVE SAMPLING PROTOCOL

Definitions

Symbol/Number	Definition	Example

Day	Day of the month	'3' – the third day of the month. Example – 3rd May
Number	Slide number from the start of the day	'2' – the second slide of the day
m	Middle slide of the day, rounded up	17 slides prepared on the day.<br>m = 17/2 = 8.5<br>Rounded up = slide 9
Last	Last slide of the day	17 slides prepared on the day.<br>Last slide = slide 17
m +/- number	Slide number counted forward or back from middle	'm – 2' = 2nd slide before middle<br>If middle slide = 9<br>m-2 = slide 7
Last - number	Slide number counted back from the last slide	'Last – 3' = 4th last slide of the day<br>If last slide = 17<br>Last – 3 = slide 14

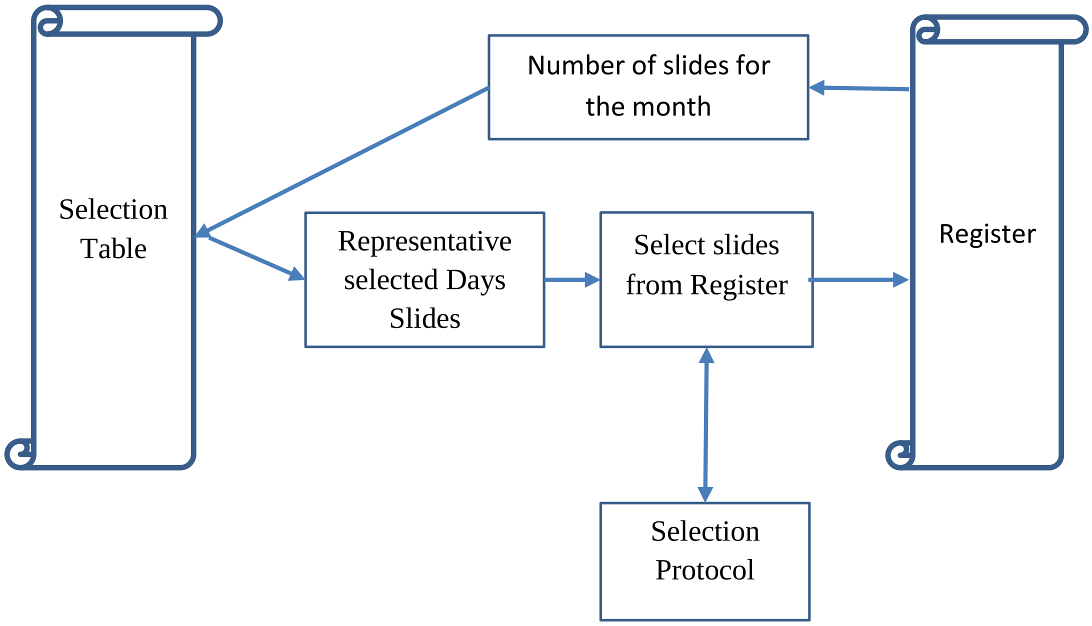

Protocol

1. **Only select Negative Blood Films using this protocol** (all positive blood films must be separately selected)

2. Use the **Representative Sampling Table** to select QC blood films **<u>from the Register Book</u>**.

   2.1. Do not select blood films directly from the slide box.

3. Determine the number of blood films prepared during the month from the Register Book.
   Example: 385 blood films prepared during May.

4. Refer to the band of selection criteria in the Representative Sampling Table corresponding to the number of blood films prepared during the month.
   Example: For 385 slides, use the band '380-399' (this includes 385)

5. Sampling Day selection:

   5.1. From the Register Book sample QC slides from the Days given in the Representative Sampling Table
   Example: For band '380-399' the sampling days are days 3, 4, 9, 11, 15, 16, 19, 24, 25, 31

   5.1.1. For April, June, September and November – if the sampling day = 31, then use day 30.

   5.1.2. For February non-leap year – if the sampling day = 29, 30 or 31, use 28 (the last day)

   5.1.3. For February leap year – if the sampling day = 30 or 31, then use 29 (the last day)

6. Slide Number selection:

   6.1. Select the slide number for each day.

   Examples for 385 slides:
   Select the 1st slide prepared on day 3
   Select the slide immediately before the last slide prepared on day 4
   Select the 2nd slide before the middle slide on day 9

7. If the nominated blood film is Positive:

   7.1. Select one slide before or after the nominated slide.

   Examples for 385 slides:
   If the 1st slide on day 3 is Positive, then select the 2nd slide on day 3.
   If the slide immediately before the last slide prepared on day 4 is Positive, select either the last slide, or the 2nd slide before the last slide (If there were 20 slides on day 4, select slide 18 or 20).

8. If there are insufficient slides in the nominated day:

   8.1. Specific situations (385 slides used as an example)

**Blood films prepared on the nominated day**	**Action**	**Example**

0 (no slides or all slides are positive)	Change the day to the following day.<br><br>If there are no slides on the following day, select the next day	Day 4 requires the ‘Last-1’ slide. If there are no slides on day 4, then select the last-1 slide on day 5.
1	Ignore the slide description and select the slide	Day 4 requires the ‘Last-1’ slide. Select the one slide prepared on day 4.
2	If a number or a ‘m’ – select the 1st slide	Day 9 requires the m-2 slide – select the 1st slide<br><br>Day 15 requires the 5th slide – select the 1st slide
	If a ‘Last’ – select the 2nd slide	Day 4 requires the ‘Last-1’ slide – select the 2nd slide
3,4	If a number – select the 1st slide<br><br>If an ‘m’ – select the 2nd slide<br><br>If a ‘Last’ – select the last slide	Day 15 requires the 5th slide – select the 1st slide<br><br>Day 9 requires the m-2 slide – select the 2nd slide<br><br>Day 4 requires the ‘Last-1’ slide – select the 3rd slide
General	If a number, an ‘m +/- number’ or a ‘last – number’ condition cannot be met, select the closest slide	

REPRESENTATIVE SAMPLING TABLE

Slides/month											
40-59	Days	3	4	9	11	15	16	19	24	25	31
	Slide	m-1	m+1	1	Last -3	m	Last -1	m-1	m+1	2	Last
60-79	Days	2	6	8	12	14	17	20	23	26	29
	Slide	2	Last -1	m-1	m+1	3	Last	1	Last -2	m-1	m+1
80-99	Days	3	5	9	10	13	16	21	24	27	30
	Slide	m-2	m+2	2	Last -1	m-2	m	3	Last	1	Last -2
100-119	Days	1	4	7	12	13	18	21	22	27	30
	Slide	m-1	m	1	Last -1	2	Last	m	Last	m-1	m+1
120-139	Days	2	5	8	10	14	17	20	23	26	28
	Slide	1	Last -1	m-1	m+1	2	Last	m	Last	m-1	m
140-159	Days	3	4	9	11	15	16	19	24	25	31
	Slide	m-1	m+1	1	Last -3	m	Last -1	m-1	m+1	2	Last
160-179	Days	2	6	8	12	14	17	20	23	26	29
	Slide	2	Last -1	m-1	m+1	3	Last	1	Last -2	m-1	m+1
180-199	Days	3	5	9	10	13	16	21	24	27	30
	Slide	m-2	m+2	2	Last -1	m-2	m	3	Last	1	Last -2
200-219	Days	1	4	7	11	15	18	19	22	25	28
	Slide	3	Last -2	m-1	m+2	2	Last -1	1	Last	m	m+1
220-239	Days	1	4	7	12	13	18	21	22	27	30
	Slide	m-2	m	3	Last -2	2	Last - 1	m-1	m+1	1	Last
240-259	Days	2	5	8	10	14	17	20	23	26	28
	Slide	1	Last - 1	2	Last -2	m	m+2	3	Last	m-1	m+1
260-279	Days	3	4	9	11	15	16	19	24	25	31
	Slide	m-1	m+2	1	Last	2	Last - 1	m	m+1	3	Last -2
280-299	Days	2	6	8	12	14	17	20	23	26	29
	Slide	4	Last -2	m+2	m+1	1	Last	2	Last - 1	m-1	m
300-319	Days	3	5	9	10	13	16	21	24	27	30
	Slide	m-2	m+1	1	Last -3	m-1	m+2	2	Last -3	4	Last -2
320-339	Days	1	4	7	11	15	18	19	22	25	28
	Slide	5	Last - 1	m-1	m+1	1	Last	m-1	m+3	3	Last -3
340-359	Days	1	4	7	12	13	18	21	22	27	30
	Slide	5	Last - 1	1	Last	m-2	m+1	3	Last -3	m	m+2
360-379	Days	2	5	8	10	14	17	20	23	26	28
	Slide	m-1	m+1	4	Last -2	2	Last -3	m-1	m+2	1	Last - 1
380-399	Days	3	4	9	11	15	16	19	24	25	31
	Slide	1	Last - 1	m-2	m+1	5	Last -3	3	Last -5	m-1	m+2
400-419	Days	2	6	8	12	14	17	20	23	26	29
	Slide	m-4	m+1	1	Last -2	m-1	m+3	3	Last -3	4	Last
420-439	Days	3	5	9	10	13	16	21	24	27	30
	Slide	4	Last -2	m-1	m+3	2	Last	m-3	m+1	6	Last -4
440-459	Days	1	4	7	11	15	18	19	22	25	28
	Slide	m-2	m+4	3	Last -2	1	Last	5	Last -4	m-1	m+2

Slides/month											
460-479	Days	1	4	7	12	13	18	21	22	27	30
	Slide	2	Last -5	m-3	m+1	4	Last -3	m-1	m+3	6	Last - 1
480-499	Days	2	5	8	10	14	17	20	23	26	28
	Slide	m-1	m+2	1	Last -3	m	m+1	3	Last -4	6	Last
500-519	Days	3	4	9	11	15	16	19	24	25	31
	Slide	4	Last -4	m-1	m+4	2	Last -2	5	Last -6	m-3	m+1
520-539	Days	2	6	8	12	14	17	20	23	26	29
	Slide	m-2	m+3	6	Last -3	m-4	m+1	1	Last - 1	4	Last -6
540-559	Days	3	5	9	10	13	16	21	24	27	30
	Slide	7	Last -5	m-3	m+1	5	Last - 1	m-1	m+4	3	Last -3
560-579	Days	1	4	7	11	15	18	19	22	25	28
	Slide	m-1	m+4	8	Last -2	1	Last -4	4	Last	m-3	m+1
580-599	Days	1	4	7	12	13	18	21	22	27	30
	Slide	3	Last -4	m	m+4	7	Last -2	m-1	m+2	5	Last -6
600-619	Days	2	5	8	10	14	17	20	23	26	28
	Slide	6	Last -6	1	Last - 1	m-1	m+4	4	Last -4	m-3	m+1
620-639	Days	3	4	9	11	15	16	19	24	25	31
	Slide	m-1	m+5	3	Last	5	Last -5	m-3	m+1	8	Last -3
640-659	Days	2	6	8	12	14	17	20	23	26	29
	Slide	4	Last -5	6	Last -3	m-2	m+4	2	Last	m-4	m+2
660-679	Days	3	5	9	10	13	16	21	24	27	30
	Slide	7	Last -7	m-1	m+3	3	Last -4	m-4	m+1	1	Last - 1
680-699	Days	1	4	7	11	15	18	19	22	25	28
	Slide	m	m+4	5	Last -7	2	Last -4	8	Last - 1	m-2	m+2
700-719	Days	1	4	7	12	13	18	21	22	27	30
	Slide	9	Last -4	6	Last -7	m-3	m+1	m-1	m+4	1	Last
720-739	Days	2	5	8	10	14	17	20	23	26	28
	Slide	2	Last -2	m-1	m+5	4	Last -7	7	Last -4	m-3	m+1
740-759	Days	3	4	9	11	15	16	19	24	25	31
	Slide	m-2	m+4	6	Last - 1	m-4	m+2	3	Last -7	8	Last -4
760-779	Days	2	6	8	12	14	17	20	23	26	29
	Slide	9	Last -6	m	m+5	2	Last	m-3	m+3	5	Last -3
780-799	Days	3	5	9	10	13	16	21	24	27	30
	Slide	8	Last -6	3	Last	m-5	m+2	6	Last -3	m	m+7
800-819	Days	1	4	7	11	15	18	19	22	25	28
	Slide	m-1	m+4	1	Last -5	9	Last -8	m-3	m+1	5	Last -2
820-839	Days	1	4	7	12	13	18	21	22	27	30
	Slide	10	Last -8	m-3	m+1	4	Last -4	8	Last	m-1	m+5
840-859	Days	2	5	8	10	14	17	20	23	26	28
	Slide	m-2	m+6	7	Last -7	m-4	m+2	2	Last -3	9	Last
860-879	Days	3	4	9	11	15	16	19	24	25	31
	Slide	1	Last -4	m-1	m+7	8	Last - 1	6	Last - 6	m-5	m+3
880-899	Days	2	6	8	12	14	17	20	23	26	29
	Slide	m-3	m+7	5	Last	7	Last -8	m-7	m+3	3	Last -3

Slides/month											
900-919	Days	3	5	9	10	13	16	21	24	27	30
	Slide	4	Last	10	Last -3	m-1	m+6	6	Last -7	m-4	m+1
920-939	Days	1	4	7	11	15	18	19	22	25	28
	Slide	5	Last -9	m-7	m+2	2	Last - 6	m-3	m+6	8	Last -3
940-959	Days	1	4	7	12	13	18	21	22	27	30
	Slide	m-1	m+6	4	Last - 6	m-4	m+3	9	Last -9	7	Last - 1
960-979	Days	2	5	8	10	14	17	20	23	26	28
	Slide	3	Last -7	m-3	m+7	10	Last -10	6	Last -3	m-6	m+2
980-999	Days	3	4	9	11	15	16	19	24	25	31
	Slide	m-2	m+5	8	Last -10	11	Last - 1	m-5	m+3	2	Last -5

DATA ENTRY FORMS

Laboratory name		Date QC performed	
Month (Round)		Cross-checker name	

MACROSCOPIC – Positive Blood Films

Score 1 point for each correct parameter

POSITIVE THICK FILM MACROSCOPIC	Slides																				
	1	2	3	4	5	6	7	8	9	10	11	12	13	14	15	16	17	18	19	20	Total score
Number of slides examined																					
Labelling – correct - fully and clearly labelled																					
Size – correct - same shape and size as template																					
Wash-away – no parts of the thick film washed away																					
Number of slides able to be further examined																					
Thickness - consistent with the correct blood volume																					
Uniformly spread																					
Not fixed by methanol																					
Not cracked due to overheating																					

MACROSCOPIC – Negative Blood Films

Score 1 point for each <u>correct</u> parameter

NEGATIVE THICK FILM MACROSCOPIC	Slides										
	1	2	3	4	5	6	7	8	9	10	Total score
Number of slides examined											
Labelling – correct - fully and clearly labelled											
Size – correct - same shape and size as template											
Wash-away – no parts of the thick film washed away											
Number of slides able to be further examined											
Thickness - consistent with the correct blood volume											
Uniformly spread											
Not fixed by methanol											
Not cracked due to overheating											

MICROSCOPIC – Positive Blood Films

Score 1 point for each correct parameter

POSITIVE THICK FILM MICROSCOPIC	Slides																				
	1	2	3	4	5	6	7	8	9	10	11	12	13	14	15	16	17	18	19	20	Total score
Number of slides examined																					
Staining																					
Not stained uniformly blue																					
No stain precipitate																					
No excessive artefacts																					
No bacterial contamination																					
No fungal contamination																					
Positive/Negative																					
Crosschecked as positive																					
Species																					
Species identified by reference laboratory	Pf				Pm				Po												
	w		s		w		s		w		s										
Total Pf-only																					
Total Pf+Pm																					
Total Pf+Po																					
Total Pf+Pm+Po																					
Total Pm-only																					
Total Po-only																					

MACROSCOPIC – Negative Blood Films

Score 1 point for each correct parameter

**NEGATIVE THICK FILM MICROSCOPIC**	Slides										
	1	2	3	4	5	6	7	8	9	10	Total score
Number of slides examined											
Staining											
Not stained uniformly blue											
No stain precipitate											
No excessive artefacts											
No bacterial contamination											
No fungal contamination											
Positive/Negative											
Crosschecked as negative											
Species											
If positive species identified:	Pf		Pm		Po						
	w	s	w	s	w	s					
Total Pf-only											
Total Pf+Pm											
Total Pf+Po											
Total Pf+Pm+Po											
Total Pm-only											
Total Po-only											
