# Supplementary figures and images for: Implementation of external quality assessment of microscopy for improved parasite detection and confirmatory diagnosis of malaria in Tanzanian Military health facilities
Source: BMC Res Notes. 2020 Sep 18;13:447. doi: 10.1186/s13104-020-05290-0 (PMC7501635; doi:10.1186/s13104-020-05290-0)

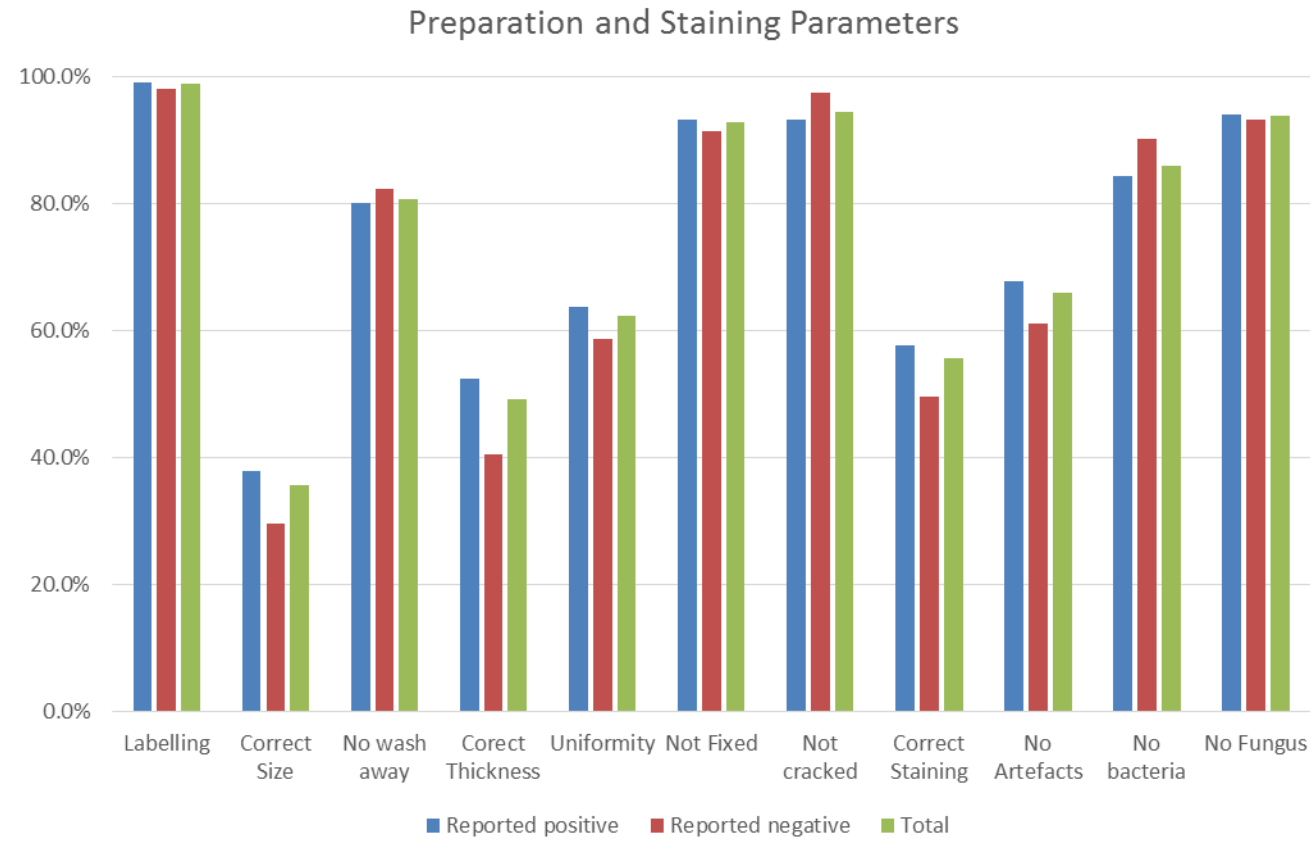

Additional file 2: Figure S2. Preparation and staining parameters of blood smears on the slide

Supplement: Supplementary file 2 — Additional file 2: Figure S2. Preparation and staining parameters of blood smears on the slide. [file 13104_2020_5290_MOESM2_ESM.pdf]

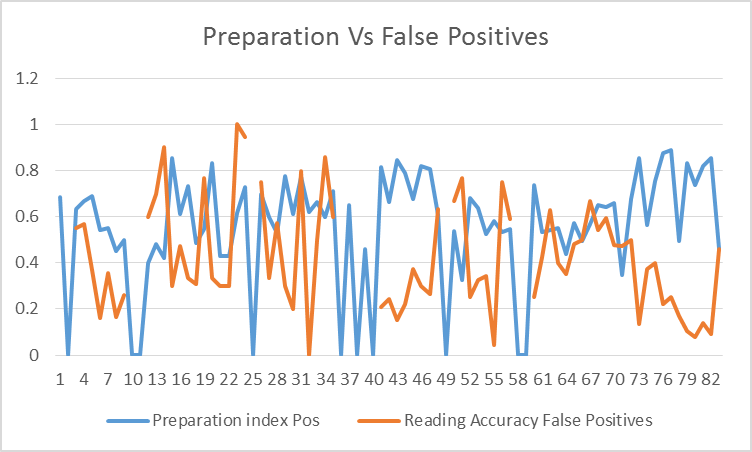


**Additional file 3**: figure S3. A graph showing trend of false positives versus preparation quality

Supplement: Supplementary file 3 — Additional file 3: Figure S3. A graph showing trend of false positives versus preparation quality. [file 13104_2020_5290_MOESM3_ESM.docx]

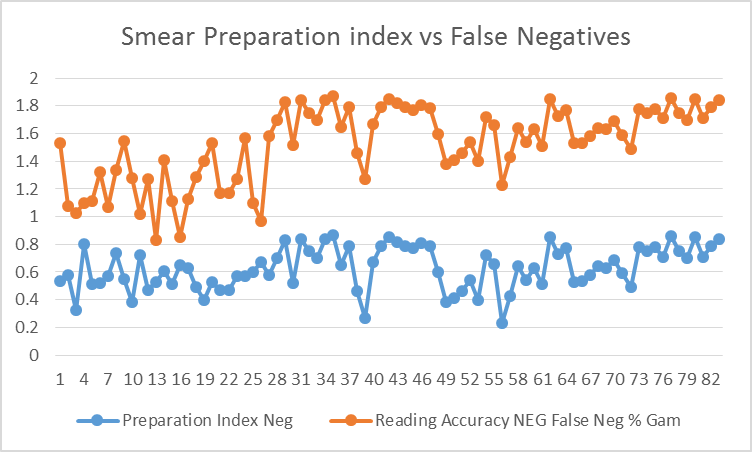
 **Additional file 2: Figure s1.** A graph showing trend of false negatives versus preparation quality

Supplement: Supplementary file 4 — Additional file 4: Figure S4. A graph showing trend of false negatives versus preparation quality. [file 13104_2020_5290_MOESM4_ESM.docx]
